# Learning curve of single-port robot-assisted simple prostatectomy: a risk-adjusted CUSUM analysis

**DOI:** 10.1007/s11701-026-03200-3

**Published:** 2026-02-11

**Authors:** Lorenzo Santodirocco, Luca A. Morgantini, Marwan Alkassis, Alexandru Turcan, Flavia Tamborino, Filippo Carletti, Simone Crivellaro

**Affiliations:** 1 Department of Urology, University of Illinois at Chicago, Chicago, IL USA; 2 Department of Maternal-Infant and Urological Sciences, Sapienza University of Rome Umberto I Hospital, Rome, Italy; 3 Department of Oncology, Division of Urology, University of Turin, San Luigi Gonzaga Hospital, Orbassano, Italy; 4 Department of Medical Oral and Biotechnological Science, Università degli Studi “G. D’Annunzio” of Chieti, Chieti, Italy; 5 Department of Surgery, Oncology and Gastroenterology, Urologic Unit, University of Padova, Padua, Italy

**Keywords:** Cumulative sum (CUSUM), Learning curve, Single port, Robot-assisted surgery, Simple prostatectomy, Benign prostatic hyperplasia (BPH)

## Abstract

Purpose: To evaluate the learning curve of robotic-assisted simple prostatectomy (RASP) using a transvesical approach and to identify the impact of patient-related factors, such as prostatic volume and Body Mass Index (BMI), on operative efficiency. Materials and methods: A retrospective analysis of 103 consecutive RASP procedures performed by a single surgeon was conducted. A multiple linear regression model was developed to calculate predicted operative times adjusted for prostatic volume and BMI. Risk-adjusted Cumulative Sum (RA-CUSUM) analysis was applied to the residuals to characterize the learning curve. Perioperative outcomes were compared across the identified phases using the χ² test or Fisher’s exact test for categorical variables and one-way ANOVA or the Kruskal-Wallis test for continuous variables. Results: Both prostatic volume (*p* < 0.001) and BMI (*p* = 0.029) were independent predictors of operative duration. The RA-CUSUM analysis identified a learning phase (cases 1–33), a proficiency phase (34–73), and a consolidation phase (74–103). Significant improvements were observed across phases for median operative time (210 vs. 176 vs. 190 min, *p* = 0.011) and median length of stay (11 vs. 9 vs. 9 h, *p* < 0.001). No significant differences were found regarding complications or functional outcomes. Conclusions: Proficiency in robotic transvesical simple prostatectomy is achieved after approximately 33 cases, with technical consolidation occurring after the 75th procedure. The transvesical approach provides direct access that may mitigate the technical challenges associated with high BMI, although prostatic volume remains the primary driver of operative duration.

**Supplementary Information:**

The online version contains supplementary material available at 10.1007/s11701-026-03200-3.

## Introduction

Benign prostatic hyperplasia (BPH) remains a leading cause of lower urinary tract symptoms (LUTS) in aging men, with surgical intervention recommended for those with large volume glands or refractory symptoms [1]. While open simple prostatectomy was historically the gold standard for prostates exceeding 80–100 mL, the last decade has seen a significant shift towards minimally invasive techniques to reduce perioperative morbidity [2]. Robot-assisted simple prostatectomy (RASP) has emerged as a robust alternative, offering comparable functional outcomes to open surgery with the benefits of reduced blood loss and shorter hospital stays [2, 3].

The introduction of the Da Vinci SP^®^ (Single-Port) robotic platform has further refined this approach, allowing for a pure transvesical access that minimizes peritoneal violation and optimizes postoperative recovery [4, 5]. However, the transition to this novel technology requires a specific surgical expertise. While the safety and feasibility of SP-RASP have been documented, detailed assessments of its learning curve using risk-adjusted methodologies are still limited [6]. Understanding the transition from proficiency to technical consolidation is essential for the widespread adoption of this technique and for optimizing surgical training protocols. We used one of the most frequently used learning curve assessment in the surgical field, the cumulative sum curve (CUSUM) of continuous variables, such as operative time (OT) and estimated blood loss (EBL) [7] to understand the number of cases required for a surgeon to achieve proficiency, which in turn is a key determinant of better patient outcomes. Furthermore, understanding these trends for a novel procedure like transvesical SP-RASP is essential to guide structured training programs, establish realistic performance expectations, and ensure informed decision-making regarding patient safety during the initial stages of technology adoption [8].

## Materials and methods

### Patient selection

In this Institutional Review Board (IRB) approved study, we retrospectively reviewed the records of the first 103 consecutive patients who underwent Single Post Robot-Assisted Simple Prostatectomy (SP-RASP) performed by a single surgeon between January 2020 and June 2025. The primary surgeon already had > 10 years of experience and > 100 cases of multi-port RASP. All procedures were performed using a transvesical approach. Patients with a history of prostate cancer or previous major bladder surgery were excluded. The study was conducted in accordance with the Declaration of Helsinki.

### Surgical procedure

The technique for trans SP-RASP has been previously described [9]. Briefly, following transvesical access, the ureteral orifices were identified bilaterally, and the planned enucleation plane was circumferentially marked. Adenoma enucleation was initiated anteriorly at the bladder neck and proceeded circumferentially toward the prostatic apices, with particular attention to ureteral orifice preservation. After cold transection of the urethra, intravesical pressure was reduced to facilitate meticulous hemostasis. Reconstruction was limited to a posterior mucosal advancement, without complete circumferential vesicourethral anastomosis, to preserve anatomical orientation and optimize postoperative recovery. The procedure concluded with catheter placement and layered bladder closure.

In the present series, the surgical steps remained consistent with the standardized approach, ensuring a uniform baseline for assessing the learning curve and operative efficiency. In all cases, a 5-mm assistant port was routinely used for suction. However, since June 2021, our institution has implemented a standardized same-day discharge (SDD) pathway for most of the patients undergoing robotic single-port urologic surgery, except for those procedures that mandate inpatient admission.

### Statistical analysis

All statistical analyses were performed using IBM SPSS Statistics version 30.0.0.0 (IBM Corp., Armonk, NY, USA). Continuous variables were reported as mean and standard deviation (SD) for normally distributed variables, or median and interquartile range (IQR) for non-normally distributed data. Categorical variables were reported as frequencies and percentages. Normality of distribution was assessed using the Shapiro–Wilk test.

We chose OT to build our CUSUM learning curve as it represents a continuous, sensitive surrogate of technical efficiency, less influenced by institutional protocols than length of stay (LOS) or post-operative complications. To account for surgical difficulty, the OT was risk-adjusted using a multiple linear regression model. The predictive power of the model was assessed using the adjusted R-squared. Multicollinearity was assessed using the Variance Inflation Factor (VIF).

The learning curve was analysed using the Risk-Adjusted Cumulative Sum (RA-CUSUM) method. The RA-CUSUM was calculated by cumulatively summing the residuals derived from the regression model.

Based on the CUSUM plot profile, we divided the surgical curve into three distinct phases:

1. Learning phase: characterized by a positive slope (increasing cumulative time).
2. Proficiency phase: indicated by a downward slope after the highest peak, representing increasing efficiency.
3. Consolidation phase: identified by a stabilization of the curve around a plateau.

Inter-phase comparisons for perioperative outcomes were performed using the χ² test or Fisher’s exact test for categorical variables and one-way ANOVA or the Kruskal-Wallis test for continuous variables. Statistical significance was considered at *p* < 0.05.

## Results

### Study cohort characteristics

The baseline characteristics of the 103 patients included in the study are summarized in Table 1. The median age of the cohort was 67 years (Standard Deviation, SD ± 8), and the mean Body Mass Index (BMI) was 28.2 Kg/m2 (SD ± 5.9). Forty-two patients (40.8%) were classified as ASA II, while 61 patients (59.2%) had an ASA III score. A history of previous benign prostatic hyperplasia (BPH) surgery was reported in 11 patients (10.7%), including 7 who had undergone prior Transurethral Resection of the Prostate (TURP). The cohort exhibited a substantial prostatic burden, as reflected by a median preoperative prostate volume of 110 g (IQR: 85–153). The median Operative Time (OT) was 191 min (SD ± 49), and the median estimated blood loss (EBL) was 50 mL (IQR: 30–150). Intraoperative complications were rare, with 102 patients (99%) experiencing none. The median postoperative haemoglobin decrease was 1 g/dL (IQR: 0–2). Continuous Bladder Irrigation (CBI) was not required in 98 cases (95.1%). Total median opioid dose was 60 (IQR: 39–75) MME (Morphine Milligram Equivalent). Most of the patients were discharged on the same day of the procedure, with a median LOS of 9 h (IQR: 8–11 h).

**Table 1:** Demographic and perioperative characteristics of the study cohort

Number	103
Age (years), mean ± SD	67 ± 8
BMI (Kg/m^2^), mean ± SD	28.2 ± 5.9
ASA score	
II	42 (40.8%)
III	61 (59.2%)
Previous BPH surgery	
No	92 (89.3%)
Yes	11 (10.7%)
Prostate size (g), median (IQR)	110 (85–153)
Operative time (min), mean ± SD	191 ± 49
EBL (ml), median (IQR)	50 (30–150)
Intraoperative complications, n° (%)	
No	102 (99%)
Yes	1 (1%)
Hb drop (g/dl), median (IQR)	1 (0–2)
CBI, n° (%)	
No	98 (95.1%)
Yes	5 (4.9%)
Total opioid dose POD0 (MME), median (IQR)	60 (39–75)
LOS (hours), median (IQR)	9 (8–11)

IQR, interquartile range; SD, standard deviation; BMI, body mass index; ASA score, American Society of Anesthesiologists; BPH, benign prostatic hyperplasia; EBL, estimated blood loss; CBI: continuous bladder irrigation; POD, post-operative day; MME, morphine milligram equivalent; LOS, length of stay

Postoperative outcomes of the study population are detailed in Table 1 of the Supplementary Material.

### Multiple linear regression

Before the RA-CUSUM analysis, a multiple linear regression model was performed to adjust for surgical difficulty. We included only preoperative factors in the regression model to avoid confounding with intraoperative performance indicators. The model was robust, with an adjusted R-squared of 0.46, identifying prostatic volume (*p* < 0.001) and BMI (*p* = 0.029) as independent predictors of operative time (OT). The lack of significant multicollinearity (VIF = 1.089) confirmed the stability of the adjustment.

### RA-CUSUM learning curve and phases comparison

**Fig. 1:**
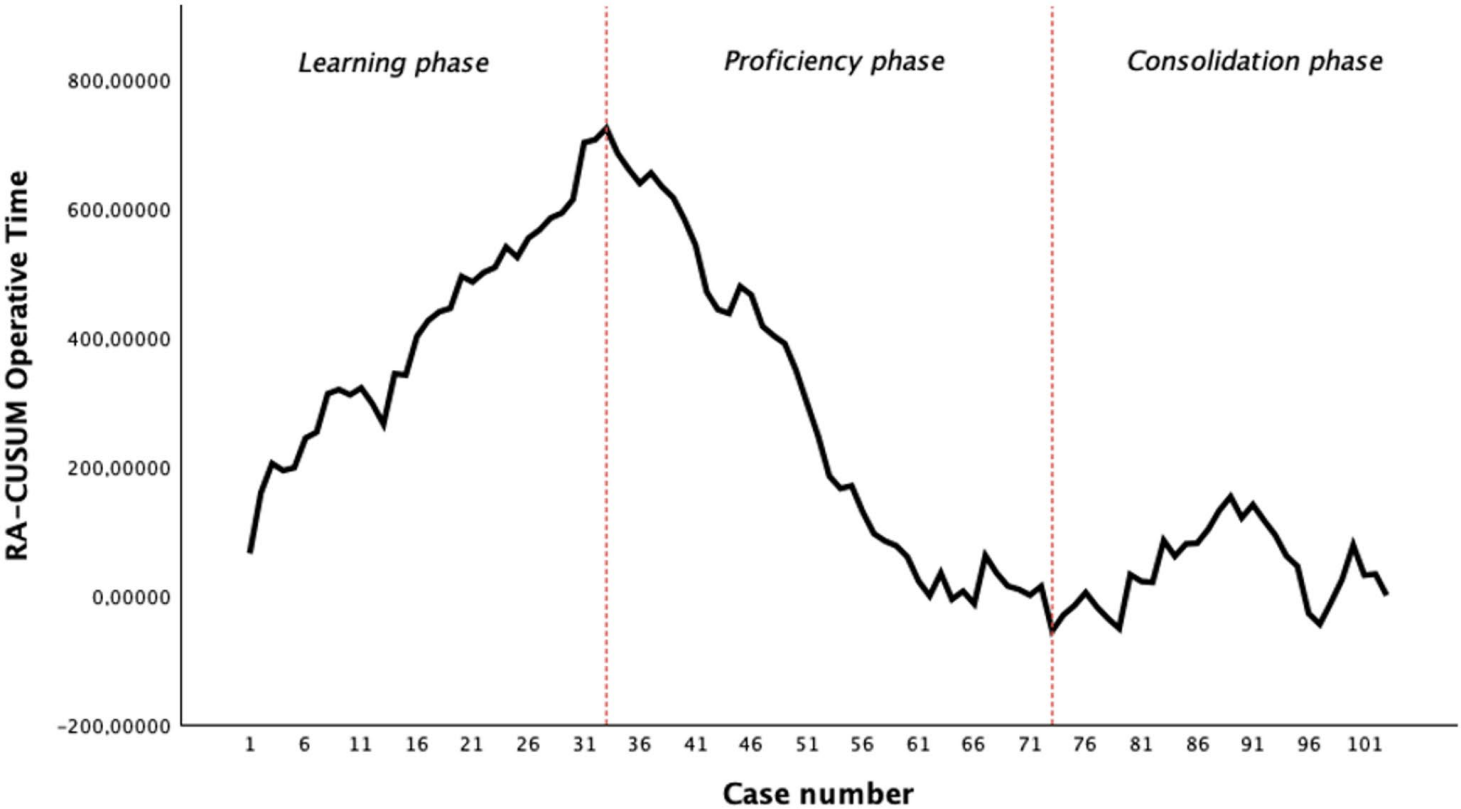
RA-CUSUM analysis for operative time

The RA-CUSUM analysis for operative time (OT) is illustrated in Fig. 1. The model, adjusted for prostatic volume and BMI, demonstrated that the surgeon’s performance followed a typical three-phase learning process.

The first phase (“learning phase”, cases 1–33) was characterized by an upward slope in the RA-CUSUM plot, reaching its highest peak at the 33rd procedure. This peak likely represents the transition from initial familiarization with the single-port transvesical approach to the consolidation of the most technically demanding steps of the procedure. During this stage, the mean OT was 210 ± 40 min, and the median LOS was 11 h.

The second phase (“proficiency phase”, cases 34–75) showed a consistent downward trend, indicating that the observed OT was lower than the model-predicted time. The gradual nature of this decline suggests progressive gains in operative efficiency rather than an abrupt improvement. In this phase, we observed a statistically significant reduction in both mean OT (176 ± 45 min, *p* = 0.011) and median LOS (9 h, *p* < 0.001) compared to the initial phase.

Finally, a third phase (“consolidation phase”, cases 76–103) was identified, where the curve reached a plateau near the zero-intercept, suggesting a stabilization of surgical performance. Although a slight increase in mean OT was noted (190 ± 57 min), this phase was characterized by the highest efficiency in terms of LOS and stable perioperative safety.

No significant differences in baseline features were observed among the learning curve phases, supporting that the variations in operative time identified by the RA-CUSUM analysis were primarily attributable to increasing surgical experience rather than changes in patient selection over time. Moreover, the case acquisition rate remained stable across learning phases.

The comparison of perioperative outcomes across these phases (Table 2) validates the transition points identified by the RA-CUSUM plot. While efficiency metrics (OT and LOS) significantly improved as experience accrued, safety indicators, such as EBL (*p* = 0.312), 90-day complication rates (*p* = 0.545), and 6-month continence (*p* = 0.619), remained stable across the entire cohort. This suggests that the transvesical SP-RASP technique is reproducible and safe from the earliest stages of adoption, with surgical consolidation primarily impacting procedural speed and post-operative recovery.

**Table 2:** Comparison of perioperative outcomes between different phases for SP-RASP

Number (%)	Learning phase	Proficiency phase	Mastering phase	*P* value
	33 (32%)	40 (38.9%)	30 (29.1%)	
Operative time, mean ± SD	210 ± 40	176 ± 45	190 ± 57	**0.011**
EBL, median (IQR)	115 (40–90)	90 (40–150)	50 (30–100)	0.312
Intraop. blood transfusion, n° (%)				
No Yes	33 (100%) 0	40 (100%) 0	30 (100%) 0	NA
Intraoperative complication, n° (%)				
No Yes	32 (97.0%)	40 (100%)	30 (100%)	0.343
	1 (3%)	0	0	
90-days postop. complications, n° (%)				
No Yes	25 (75.8%)	30 (75%)	23 (77%)	0.545
	8 (24.2%)	10 (25%)	7 (23%)	
Readmission, n° (%)				
No Yes	33 (100%)	38 (95%)	27 (90%)	0.182
	0	2 (5%)	3 (10%)	
CD, n° (%)				
1 2 3	2 (40%)	2 (20%)	2 (22.2%)	0.650
	3 (60%)	8 (80%)	6 (66.7%)	
	0	0	1 (11.1%)	
LOS (hours), median (IQR)	11 (9–28)	9 (8–10)	9 (8–9)	**< 0.001**
Hb drop (g/dl), median (IQR)	1 (0–2)	1 (1–2)	1 (1–2)	0.981
CBI, n° (%)				
No Yes	31 (93.9%)	38 (95%)	29 (100%)	0.426
	2 (6.1%)	2 (5%)	0	
Pain POD0 - VAS, n° (%)				
0–3 4–7	25 (75.8%)	32 (80%)	18 (60%)	0.053
	8 (24.3%)	8 (20%)	12 (40%)	
No narcotics pathway, n° (%)				
No Yes	20 (71.4%)	25 (62.5%)	15 (55.6%)	0.472
	8 (28.6%)	15 (37.5%)	12 (44.4%)	
SUI, n° (%)				
No Yes	32 (97%)	39 (97.5%)	30 (100%)	0.649
	1 (3%)	1 (2.5%)	0	
UUI, n° (%)				
No Yes	32 (97%)	38 (95%)	27 (90%)	0.479
	1 (3%)	2 (5%)	3 (10%)	
Continence at 3 months, n° (%)				
No Yes	1 (3%)	3 (7.5%)	1 (3.4%)	0.619
	32 (97%)	37 (92.5%)	28 (96.6%)	
Continence at 6 months, n° (%)				
No Yes	0	0	0	NA
	33 (100%)	30 (100%)	6 (100%)	
% removed prostate, mean (SD)	57 (15)	57 (14)	56 (16)	0.276

SP-RASP, single-port robotic-assisted simple prostatectomy; IQR, interquartile range; SD, standard deviation; EBL, estimated blood loss; CD, Clavien-Dindo; LOS, length of stay; CBI, continuous bladder irrigation; POD, post-operative day; VAS, visual analog scale; SUI, stress urinary incontinence; UUI, urgency urinary incontinence

## Discussion

The transition from multiport robotic systems to the Da Vinci SP^®^ platform represents a significant technical evolution in simple prostatectomy. Our results confirm that while the SP system maintains the core principles of robotic surgery, it introduces a specific learning curve related to its unique instrumentation and narrower working space [10, 11]. In our series, the RA-CUSUM analysis identified a proficiency threshold at the 33rd case. The peak observed at the 33rd case likely reflects the point at which basic procedural steps are acquired, while efficiency in the most technically demanding components, such as circumferential enucleation, apical dissection, and hemostasis, has not yet been fully optimized. The subsequent prolonged descending phase suggests a gradual and continuous refinement of operative efficiency, consistent with the technical complexity of the transvesical SP-RASP approach. Rather than an abrupt improvement, efficiency appears to improve incrementally as multiple procedural elements are progressively optimized. We observed no significant differences in safety outcomes across learning phases. This reinforces the previous findings, suggesting that SP-RASP is a safe and reproducible alternative even during the early stages of adoption [5, 12].

A central finding of our study is the role of patient-specific variables in determining operative efficiency. Through our risk-adjusted model, we confirmed that prostatic volume is the primary driver of operative time (*p* < 0.001), a finding that aligns with the established literature on both multiport and open simple prostatectomy [13, 14]. However, the impact of BMI (*p* = 0.029) deserves particular attention. While high BMI is often cited as a challenge in traditional robotic surgery due to port placement and bowel interference [15], the transvesical approach utilized in our study may mitigate some of these difficulties. By entering the bladder dome directly, the surgeon avoids the peritoneal cavity, potentially neutralizing the negative impact of visceral obesity on surgical access [5, 16]. This explains why, despite being a significant predictor, the impact of BMI remained secondary to gland volume in our regression analysis.

The most significant clinical improvement identified in our transition from the learning to the proficiency phase was the reduction in hospital stay (LOS) from a median of 11 to 9 h (*p* < 0.001), as some of the very first cases from the adoption of the technique ended up not being discharged the same day of the procedure. However, this trend should be interpreted in the context of the implementation of a standardized SDD pathway during the study period, as mentioned previously. This optimization of postoperative care is a hallmark of the SP platform. As noted by Ganesan et al. [17], the single-site access is associated with reduced postoperative pain and lower narcotic requirements, which likely facilitates earlier mobilization and discharge. Our data suggest that as the surgeon improves the transvesical technique, the overall recovery pathway becomes more streamlined.

Finally, the identification of a “consolidation phase” highlights the importance of long-term performance monitoring. As emphasized by Lin et al. [18], CUSUM analysis provides a more granular view of surgical evolution than simple mean comparisons.

Interestingly, our RA-CUSUM analysis identified a slight increase in mean operative time during the consolidation phase (190 min) compared to the proficiency phase (176 min). This ‘plateau-like’ oscillation is frequently described in robotic surgery literature [10, 19] and can be attributed to several factors. As surgical proficiency is consolidated, surgeons often feel more confident in tackling cases with higher anatomical complexity or larger median lobes, which may inherently require more meticulous dissection. Furthermore, during the later stages of the learning curve, more emphasis may be placed on refined haemostasis and precision in the trigonalization step to optimize long-term functional outcomes, even at the cost of a few additional operative minutes. Importantly, this slight temporal increase did not compromise safety, as evidenced by the stable complication rates and blood loss across the phases.

Our study is not without limitations. First, as discussed in the methodological critique by Woodall et al. [20], the retrospective use of CUSUM to identify learning phases can be subject to selection bias, as the ‘peaks’ in the curve are identified post-hoc without formal hypothesis testing for change-points. We addressed this by validating the identified phases through a direct comparison of perioperative outcomes, which confirmed a clinical shift after the 33rd case. Second, the validity of a risk-adjusted CUSUM (RA-CUSUM) depends heavily on the underlying regression model. Although our model included key preoperative factors like prostate volume and BMI, achieving an adjusted R-squared of 0.46, it cannot account for all unmeasured intraoperative variables, such as previous prostatic inflammation or specific pelvic anatomy, that might influence surgical time. Moreover, this is a single-surgeon study, whose learning curve reflects his experience and prior expertise in multiport robotic simple prostatectomy. This background almost certainly contributed to a shorter and smoother learning curve with the single-port platform. Therefore, the identified proficiency threshold should not be interpreted as directly applicable to surgeons without prior robotic experience or those at the beginning of their robotic training. However, this study provides a transparent benchmark for the adoption of the robotic transvesical approach. Lastly, due to the short follow-up, we could not compare long-term outcomes between the different phases. Another methodological consideration relates to the intrinsic limitations of CUSUM-based learning curve analyses. While CUSUM is a powerful tool for tracking performance over sequential cases, it does not explicitly account for temporal spacing between procedures or variations in case acquisition rate. As highlighted in prior methodological literature [20], differences in surgical frequency over time may influence the apparent slope and inflection points of learning curves. In the present study, however, the case acquisition rate remained stable throughout the study period, with a mean of 1.4 cases per month during the initial learning phase and 1.6 cases per month during the subsequent phases. No acceleration or reduction in surgical frequency was observed over time.

## Supplementary Information

Below is the link to the electronic supplementary material.

Supplementary Material 1

## Data Availability

No datasets were generated or analysed during the current study.
