# Supplementary material for: Learning curve of single-port robot-assisted simple prostatectomy: a risk-adjusted CUSUM analysis
Source: J Robot Surg. 2026 Feb 11;20(1):239. doi: 10.1007/s11701-026-03200-3 (PMC12891253; doi:10.1007/s11701-026-03200-3)
Supplement: Supplementary file 1 — Supplementary Material 1 [file 11701_2026_3200_MOESM1_ESM.docx]

Table 1. Postoperative outcomes of the study cohort

| Complication (within 90 days), n° (%)  No  Yes  Complication type, n° (%)  urine leak  UTI  hematuria  UTI and hematuria  sepsis  orchitis/epididymitis  urinary retention  urine leak with sepsis |  | 79 (76.7%)  24 (23.3%)  1 (4.2%)  6 (25%)  3 (12.5%)  6 (25%)  1 (4.2%)  4 (16.7%)  2 (8.3%)  1 (4.2%) |
| --- | --- | --- |
| CD, n° (%) |  |  |
| 1 |  | 6 (25%) |
| 2 |  | 17 (70.8%) |
| 3 |  | 1 (4.2%) |
| Readmission (within 90 days), n° (%) |  |  |
| No |  | 98 (95.1%) |
| Yes |  | 5 (4.9%) |
| SUI, n° (%) |  |  |
| No  Yes |  | 101 (98.1%)  2 (1.9%) |
| UUI, n° (%) |  |  |
| No  Yes |  | 97 (94.2%)  6 (5.8%) |
| Continence at 3 months, n° (%)  No  Yes |  | 5 (4.9%)  97 (95.1%) |
| Continence at 6 months, n° (%)  No  Yes |  | 0  103 (100%) |
| Time to catheter removal (days), median (IQR) |  | 5 (4-7) |
| % removed prostate, median (IQR) |  | 56 (48-67) |
| Follow up length (weeks), median (IQR) |  | 30 (26-32) |

SD, standard deviation; IRQ, interquartile range; UTI, urinary tract infection; CD, Clavien-Dindo; SUI, stress urinary incontinence; UUI, urgency urinary incontinence.
